# SMSs as an alternative to provider-delivered care for unhealthy alcohol use: study protocol for *Leseli*, an open-label randomised controlled trial of mhGAP-Remote vs mhGAP-Standard in Lesotho

**DOI:** 10.1186/s13063-024-08411-3

**Published:** 2024-09-02

**Authors:** Jennifer M. Belus, Natalie E. Johnson, Grace H. Yoon, Nadine Tschumi, Malebanye Lerotholi, Irene Falgas-Bague, Tristan T. Lee, Pearl Letsoela, Jessica F. Magidson, Alain Amstutz, Niklaus D. Labhardt

**Affiliations:** 1 Division of Clinical Epidemiology, Department of Clinical Research, University Hospital Basel, Basel, Switzerland; 2 University of Basel, Basel, Switzerland; 3 SolidarMed, Partnerships for Health, Maseru, Lesotho; 4 Swiss Tropical and Public Health Institute, Basel, Switzerland; 5 Ministry of Health, Maseru, Lesotho; 6 Department of Psychology, University of Maryland, College Park, USA; 7 Oslo Centre for Biostatistics and Epidemiology, Oslo University Hospital, Oslo, Norway; 8 Bristol Medical School, Population Health Sciences, University of Bristol, Bristol, UK; 9 Center for Substance Use, Addiction & Health Research (CESAR), University of Maryland, College Park, United States

**Keywords:** SMS, mhGAP, Problem alcohol use, Digital intervention, Randomised controlled trial, Lesotho

## Abstract

**Background:**

The World Health Organization’s (WHO) Mental Health Gap Action Programme (mhGAP) is a validated intervention that can be provided by non-specialised healthcare workers to individuals with unhealthy alcohol use. However, it typically requires several in-person sessions at a health facility, which may limit its feasibility and effectiveness in remote settings. This trial compares mhGAP-Standard, a 4 to 6 in-person session intervention, to mhGAP-Remote, a 1 in-person session intervention followed by 8 week of short message service (SMS) in Lesotho. We hypothesise that mhGAP-Remote is superior to mhGAP-Standard in reducing alcohol use (as detailed by the primary and secondary outcomes below).

**Methods:**

This is a two-arm randomised open-label multicentre superiority trial. Participants allocated to mhGAP-Standard receive 4 in-person sessions using motivational interviewing, identifying triggers, and alternative behaviours, with the option of two additional booster sessions. Participants in the mhGAP-Remote arm receive 1 in-person session covering the same content, followed by standardised SMSs over 8 weeks that reinforce intervention content. Non-specialist providers deliver the intervention and receive weekly supervision. Adults (*N*_planned_ = 248) attending participating health facilities for any reason and who meet criteria for unhealthy alcohol use based on the Alcohol Use Disorders Identification Test ([AUDIT] score ≥ 6 for women, ≥ 8 for men) are individually randomised to the two arms (1:1 allocation, stratified by participant sex and age (≥ 50 vs < 50 years old). Follow-up assessments occur at 8, 20, and 32 weeks post-randomisation. The primary outcome is change in self-reported alcohol use (continuous AUDIT score), from baseline to 8 weeks follow-up. Change in the AUDIT from baseline to 20 and 32 weeks follow-up is a secondary outcome. Change in the biomarker phosphatidylethanol (secondary), liver enzyme values in serum (exploratory), and HIV viral load (for people with HIV only; exploratory) are also evaluated from baseline throughout the entire follow-up period. A linear regression model will be conducted for the primary analysis, adjusted for the stratification factors. Three a priori sensitivity analyses for the primary outcome are planned based on per protocol treatment attendance, recovery from unhealthy alcohol use, and clinically significant and reliable change.

**Discussion:**

This trial will provide insight into feasibility and effectiveness of a shortened and primarily SMS supported version of mhGAP, which is especially relevant for settings where regular clinic attendance is a major barrier.

**Trial registration:**

clinicaltrials.gov NCT05925270. Approved on June 29th, 2023.

**Supplementary Information:**

The online version contains supplementary material available at 10.1186/s13063-024-08411-3.

## Administrative information

Note: the numbers in curly brackets in this protocol refer to SPIRIT checklist item numbers. The order of the items has been modified to group similar items (see http://www.equator-network.org/reporting-guidelines/spirit-2013-statement-defining-standard-protocol-items-for-clinical-trials/).

**Table:** 

Title {1}	SMSs as an Alternative to Provider-Delivered Care for Unhealthy Alcohol Use: Study Protocol for *Leseli,* an Open-Label Randomised Controlled Trial of mhGAP-Remote vs mhGAP-Standard in Lesotho
Trial registration {2a and 2b}.	The trial was registered at Clinicaltrials.gov: NCT05925270, on June 29th, 2023. Please refer to the clinicaltrials.gov registry for this trial at this link: https://clinicaltrials.gov/study/NCT05925270. All items from the WHO trial registration data set can be found within the protocol. See supplementary materials for the SPIRIT checklist.
Protocol version {3}	Protocol version number 1.0 (Date: 15th May, 2023)
Funding {4}	This study is funded by the Swiss National Science Foundation (PZ00P1_201690, PI: Belus).
Author details {5a}	
Name and contact information for the trial sponsor {5b}	Division of Clinical Epidemiology, Department of Clinical Research, University Hospital Basel, Totengässlein 3, 4051 Basel, Switzerland Contact: + 41612652525
Role of sponsor {5c}	The study design, collection, management, analysis, and interpretation of data, writing of the report, and the decision to submit the report for publication are the sole decision of the authors.

## Introduction

### Background and rationale {6a}

Mental health and substance use problems account for over 20% of years lived with disability globally, including in low- and middle-income countries (LMICs) [1, 2]. It is estimated that 237 million men and 46 million women suffer from alcohol use disorders [3]. Harm from alcohol use disproportionally affects lower-income drinkers [4]. In LMICs, it is estimated that over 95% of people with substance use disorders, including alcohol, do not receive treatment [5, 6]. Reasons for the large treatment gap include a lack of trained mental health providers [7], overburdened health systems [8, 9], and few resources devoted to improving provision of mental health care, including for unhealthy alcohol use [10].

Despite such barriers, several advancements in improving healthcare delivery for alcohol use and other mental health problems in LMICs have been made. Several systematic reviews and meta analyses show that non-specialist providers can be trained to deliver brief, evidence-based interventions, and that mental health and substance use problems significantly improve as a result [11–13]. The World Health Organization (WHO) developed a training and intervention guide for non-specialist providers, called the Mental Health Gap Action Programme (mhGAP), to manage mental health, neurological, and substance use problems [14]. mhGAP was designed as a flexible intervention guide and decision-making tool so that non-specialist providers could implement evidence-based interventions in their setting. It provides guidance on both behavioural and medication approaches, according to the current evidence. mhGAP as an intervention approach has been tested in 162 studies since 2017 across various clinical and research settings [15]. These studies demonstrate improved mental health knowledge, attitudes, and confidence in providers, as well as improved psychosocial outcomes, including mental health problems for service users [15].

Despite the benefits of mhGAP, there remains an estimated shortage of over 1.2 million mental health providers [16]. Additional strategies to increase access to evidence-based treatment are therefore needed. The use of technology as an alternative to provider-delivered time is an appealing and promising strategy, as interventions that utilise technology have a much larger dissemination potential than interventions that rely solely on human resources [17]. In LMICs, digital psychological interventions (primarily smartphone apps or websites) have been shown to be superior to in-person interventions for the treatment of mental health and substance use conditions in a recent meta-analysis of 22 randomised controlled trials [18]. Although promising, the use of advanced technology with fast internet connectivity is often not accessible to individuals in very low-income communities or in rural settings.

Short message service (SMS) is a feasible, simple technology approach for low-resource settings and has been successfully implemented in both high- and low-resource countries for the treatment of alcohol use in various capacities. This includes providing treatment reminders, opportunities for service users to get additional support, and delivering intervention content [19–24]. SMSs may therefore be a promising alternative strategy to deliver mhGAP intervention sessions, with the possibility of improving effectiveness because the entirety of the intervention can be delivered to service users with likely little attrition.

### Objectives {7}

The primary objective of this study is to test the effectiveness of “mhGAP-Remote”, which consists of 1 in-person mhGAP session followed by standardised SMSs for 8 weeks, compared to 4 to 6 in-person mhGAP sessions, called “mhGAP-Standard”, on self-reported alcohol use at 8 weeks post-randomisation. We hypothesise that mhGAP-Remote is superior to mhGAP-Standard. Secondary objectives include the assessment of self-reported alcohol use at other time points and phosphatidylethanol (PEth), a biological marker of alcohol use. Serum liver enzymes and HIV viral load (subsample with HIV only) are exploratory objectives.

### Trial design {8}

This study is an open-label, multicentre, two-arm individually randomised superiority trial and corresponds to a Stage III trial testing a behavioural intervention [25].

## Methods: participants, interventions, and outcomes

### Study setting {9}

The study takes place in Lesotho, a lower middle-income country located in southern Africa [26], which has one of the highest rates of HIV globally [27]. The study enrols persons attending district hospitals or primary health clinics in Butha Buthe, a primarily rural district in northern Lesotho. Butha Buthe has ten nurse-led rural health centres, one missionary hospital, and one district hospital [28]. Studies testing the use of eHealth-delivered tools for other chronic health conditions, including SMSs, are currently being evaluated in this region [29].

In Lesotho, available services for alcohol use and other mental health concerns are minimal. There is one inpatient psychiatric hospital in the capital city and short-term observation units staffed with at least one psychiatric nurse at the district hospitals for individuals experiencing acute psychiatric symptoms. Some providers working for government facilities have received mhGAP training in prior years, though the provision of mhGAP-based services is not widely utilised. Areas outside the district hospitals have limited access to mental health services, with health centres staffed by primary care nurses with no mental health training. All further mental health services from the district level are referred to the psychiatric hospital in the capital for treatment.

### Eligibility criteria {10}

#### Inclusion criteria

Participants are eligible for the study if they meet the following criteria at screening: (1) are adults (≥ 18 years old); (2) consume alcohol at a level consistent with “hazardous drinking” according to the Alcohol Use Disorders Identification Test (AUDIT) (total score of ≥ 6 for women, ≥ 8 for men); (3) have cell phone access at least half the days of the week, regular access to electricity to charge the phone, and are comfortable receiving study-specific SMSs on the phone related to alcohol use treatment; (4) willing to participate in a study focused on reducing alcohol use; (5) willing and able to regularly come to the health facility for intervention sessions during the active intervention period; (6) able to read in Sesotho or English or have a treatment supporter (e.g., family member) to read study-related materials to them; (7) willing to have the study intervention sessions audio-recorded; and (8) attends one of the study clinics and intend to remain at the same clinic for 8 months.

#### Exclusion criteria

Participants are excluded from the study if they meet any of the following criteria: (1) high-risk alcohol use that warrants medical management; (2) known brain pathology (e.g., brain tumour), history of epilepsy, or history of delirium, as these factors increase risk for withdrawal symptoms that require medical management; (3) untreated major mental illness that interferes with study participation, such as psychosis, or mania; (4) reported pregnancy at time of enrolment; (5) currently receiving psychological treatment for alcohol use, such as behavioural therapy; (6) participation in another trial that is judged by the site investigator as non-compatible with this study; (7) unable or not willing to provide informed consent.

### Who will take informed consent? {26a}

A trained member of the study team who is fluent in both Sesotho and English and has appropriate ethical research training seeks informed consent from potential participants prior to enrolment. The study team member first seeks verbal consent prior to conducting any study screening procedures. Potential participants are provided with a hard copy of the consent form to review (see supplementary materials for the model consent form to be used). Then, the study team member reviews each section of the consent form with participants individually, explaining the nature of the study, its purpose, the procedures involved, expected duration, potential risks and benefits, and remuneration. The voluntary nature of participation in the study is emphasised. Participants have the opportunity to ask questions and study team members are trained to check participant understanding of the study procedures. Participants who are illiterate complete the informed consent process in the presence of a witness and provide their thumbprint (rather than signature) to agree to the study terms. 

### Additional consent provisions for collection and use of participant data and biological specimens for ancillary studies {26b}

Additional measures related to psychosocial outcomes and feedback on the intervention were administered to participants as part of the main trial data collection but will be analysed in subsequent publications. The trial consent form included these aspects of data collection; as such, there are no additional consent provisions related to subsequent analyses.

## Interventions

### Explanation for the choice of comparators {6b}

The comparator in this study is a standardised version of mhGAP, which is the recommended standard of care in LMICs where there is a shortage of specialised mental health care providers. The Ministry of Health in Lesotho is undertaking efforts to expand their existing mental health workforce using the psychological treatment tools provided by the WHO, including mhGAP, although this is not currently the standard of care in the country.

### Intervention description {11a}

#### mhGAP-Standard

mhGAP-Standard refers to a standardised intervention consisting of 4 in-person sessions based on the psychosocial interventions described in the WHO’s mhGAP module for substance use [15]. mhGAP is a flexible intervention guide designed to equip non-specialist providers in LMIC settings with the skills to deliver treatment for mental health and neurological conditions [14]. The intervention components are primarily focused on brief motivational interviewing, identifying triggers for use and high-risk situations, and problem-solving strategies to reduce or stop alcohol use. Participants are asked to practice skills learned in between sessions. The intervention uses a harm reduction approach, meaning that participants do not need to stop using alcohol altogether. Sessions last approximately 45–60 min each and are designed to be delivered approximately weekly, although in practice this is challenging due to scheduling difficulties and distance to the clinic. Interventionists have the option to deliver up to two additional “booster sessions” to participants who may benefit. Supplementary Table 1 provides an overview of the content covered in each session.

#### mhGAP-Remote

Participants in mhGAP-Remote receive 1 in-person session covering the core skills of mhGAP used in the mhGAP-Standard arm, followed by standardised SMSs for a period of 8 weeks that reinforce the intervention content learned. An example SMS is, “Identify your triggers. Name a person, place, or thing that makes you want to drink alcohol. Choose one and make a plan not to interact with it this week.” SMSs are sent to participants on average twice per week at standardised days and times set by the study team. A test SMS is sent to the participant’s phone prior to them leaving the first session to ensure the phone can adequately receive SMSs and that the participant understands the delivery format. Interventionists schedule a brief check-in call with participants 2 weeks after the in-person session to ensure participants are receiving the study-related SMSs and to troubleshoot any barriers to implementing skills learned. Participants are also able to request brief telephonic support from the interventionist if they struggle to implement the skills learned by reaching out to their interventionist via SMS or phone call to request this support.

To derive the standardised SMSs used in mhGAP-Remote, formative qualitative work was conducted by the study team to gather community feedback on the content of the SMSs. SMSs were drafted based on material covered in the mhGAP psychosocial interventions for substance use. A trained member of the study team conducted in-depth interviews with 28 community members, in which they gave input on the clarity, content, and utility of each SMS. Based on this feedback, the study team (comprised of Sesotho and English speakers) adapted the content of the SMSs to improve clarity as well as to fit within the 150-character limit used by the programme sending SMSs. Additional SMSs were added after the mhGAP-Remote intervention manual was finalised, as we wanted to ensure that all important content from the in-person session was reinforced via the SMSs. These additional SMSs were reviewed as a team and changes made to ensure clarity. All SMSs underwent a forward- and backward-translation process between English and Sesotho. The final list of standardised SMSs consists of 18 SMSs. See Supplementary Table 2 for a list of the intervention SMSs and timing of delivery.

#### Interventionist training and supervision

Of the two study interventionists, one has a bachelor’s degree in social work and the other in pastoral counselling. Both followed a systematic training process to learn the study interventions. First, they received a general training using the WHO’s provided mhGAP training for healthcare providers, which took place over 3 days and covered the following topics: introduction to mhGAP, essential care and psychotherapy practice, substance use, depression, and self-harm/suicide. This general training was also provided to other healthcare providers in the area as part of local capacity building. 

This general training was then followed by specific training in the intervention manuals developed for each treatment arm; this training was only provided to study interventionists. Study specific training included discussions on cultural and language appropriateness to increase cultural relevance of the material, motivational interviewing theory, following a session agenda and structure while adapting content to participant needs, and research protocol revision for special cases (e.g. participants with other mental health or chronic condition comorbidities). The training ended with two role-play practices for each treatment arm manual, which were audio-recorded. There was a total of 14 h of role-play practice for each interventionist. The first role-play practices were conducted in English to facilitate training evaluation by the main trainers. The remainder of role-play practices were conducted in Sesotho and evaluated by a Sesotho-speaking research team member who was trained on using a fidelity monitoring form to evaluate the core content of the intervention sessions as well as intervention process. Once fidelity was above 75%, interventionists were considered trained and ready to each start with pilot cases (up to 5 each) and subsequently with trial participants. Ongoing training continues with weekly group supervision sessions throughout the trial. Oversight of the training and supervision were provided by a clinical psychologist (JMB) and a psychiatrist (IFB), both with prior experience in training and supervising lay providers [30, 31].

### Criteria for discontinuing or modifying allocated interventions {11b}

Participants are informed of the voluntary nature of participation in the study and may choose to withdraw their consent to participate at any time. Participants may be referred to a higher level of care if needed for alcohol use or any other medical conditions, including experiencing symptoms of withdrawal from alcohol use that require medical management. However, these participants may continue their participation in the study if they wish and if feasible from a medical perspective. All cases of discontinuation are documented and reported according to the study adverse events procedures (see section “Adverse event reporting and harms {22}”).

### Strategies to improve adherence to interventions {11c}

An intervention manual for each study arm was developed that outlines the specific intervention techniques that must be implemented and provides specific scripts to guide the interventionists. There are also participant worksheets used to facilitate delivery of the intervention content. The study interventionists were trained specifically on the intervention treatment manuals (see section “Intervention description {11a}” for a more detailed description). We then developed a fidelity form for each session that covers each essential content component of these treatment manuals. This form consists of between seven and 24 items, depending on the session, and assesses fidelity of setting up and ending the session (e.g., setting an agenda, assigning homework), intervention components (e.g., motivational interviewing, identifying triggers for use), use of time, and effective communication skills. The interventionists self-rate their fidelity after each session using this form. A bilingual Sesotho-English speaker who has received training on both intervention manuals listens to one to two audio-recorded intervention sessions weekly (approximately 10% of all sessions) and rates the interventionist’s fidelity to the intervention. These ratings are then discussed in weekly supervision meetings.

### Relevant concomitant care permitted or prohibited during the trial {11d}

Participants are free to seek any health service they require during the study period.

### Provisions for post-trial care {30}

After conclusion of the study interventions, no additional treatment is provided. Participants in need of further treatment are referred to the district psychiatric nurse for ongoing support. Participants can continue to access the intervention worksheets and list of withdrawal symptoms necessitating medical management after the study is complete.

### Outcomes {12}

Table 1 presents the flow of recruitment and treatment, as well as the self-report and biomedically assessed measures at each time point.

**Table 1:**
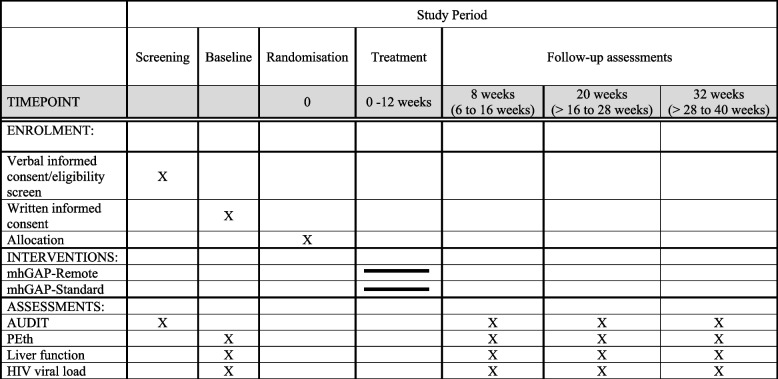
Schedule of study enrolment, interventions, and assessments

*AUDIT* Alcohol Use Disorders Identification Test, *PEth* phosphatidylethanol

#### Primary outcome

The primary outcome is mean change in self-reported alcohol use from baseline to 8 weeks follow-up (range 6–16 weeks), as measured by the continuous AUDIT score. The AUDIT is a 10-item self-report measure used extensively in LMICs to determine alcohol consumption, severity of use, symptoms of dependence, and alcohol-related problems [32, 33]. Total scores range from 0 to 40, with higher scores indicating more problematic alcohol use.

#### Secondary outcomes

Secondary outcomes include mean change in self-reported alcohol use from baseline to 20-weeks (range: >16–28 weeks) and 32 weeks (range: >28–40 weeks) follow-up, as measured by the continuous AUDIT score. In addition, we measure mean change in alcohol use according to the biomarker phosphatidylethanol (PEth) from baseline to 8 weeks (range 6–16 weeks), 20 weeks (range: >16–28 weeks), and 32 weeks (range: >28–40 weeks) follow-up. PEth is a biomarker found in the blood, with values greater than 50 ng/mL typically used to indicate unhealthy alcohol consumption [33]. This measure has been used in prior studies with participants in sub-Saharan Africa [34–37].

#### Exploratory outcomes

This study will examine change in liver serum enzymes and HIV viral load as exploratory outcomes. Specifically, change in mean serum liver enzymes aspartate aminotransferase (AST) and alanine aminotransferase (ALT) from baseline to 8 (range 6–16 weeks), 20 (range >16–28 weeks), and 32 weeks follow-up (range >28–40 weeks). Data are either extracted from participants' medical chart or collected via blood draw at the health facility, if a test result in the past 30 days is unavailable. Elevated levels above normal range indicate liver cell damage that may or may not be caused by alcohol.

For participants with HIV, change in HIV viral load will be measured dichotomously (< 50 and ≥ 50 copies/ml) from baseline to 8 (range 6–16 weeks), 20 (range >16–28 weeks), and 32 weeks (range  28–40 weeks) follow-up. Data are extracted from participants' medical chart or collected via blood draw at the health facility, if a test result in the past 30 days is unavailable. Viral load measurements < 50 copies/mL are considered virally suppressed.

### Participant timeline {13}

**Fig. 1:**
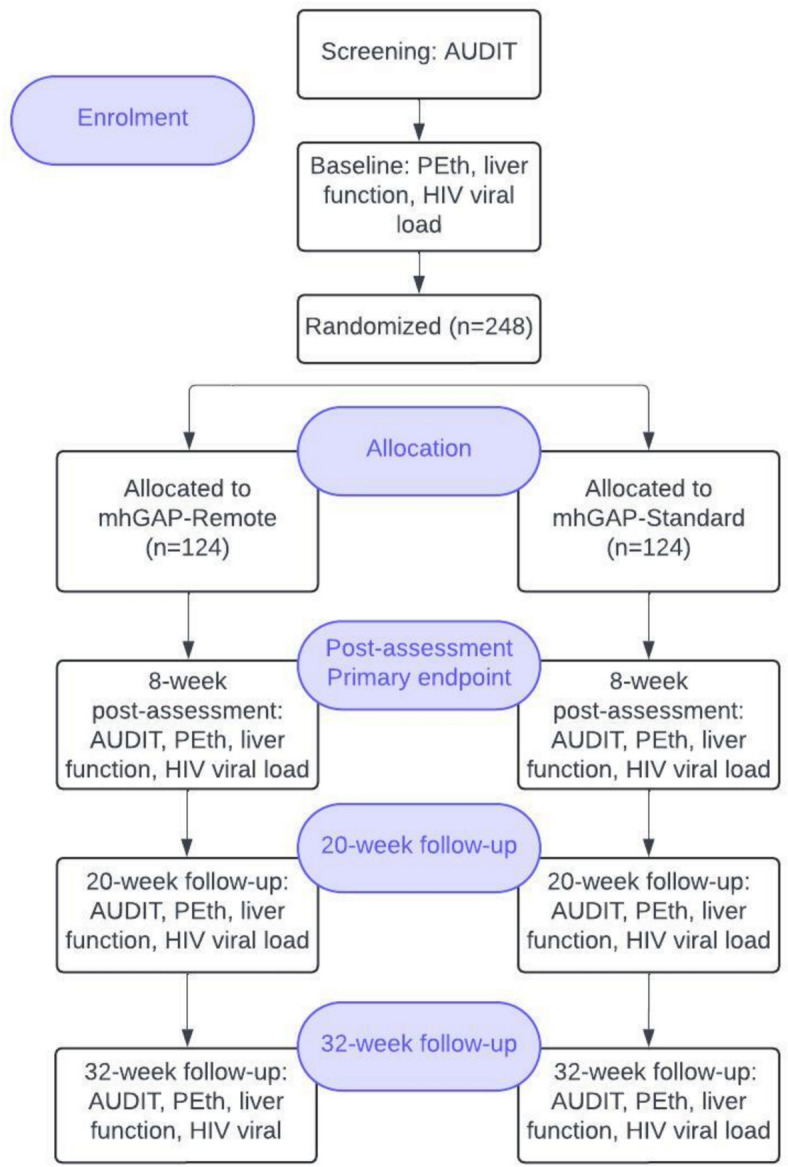
Flow chart of the trial

Study participants undergo eligibility screening and informed consent prior to taking part in the baseline assessment. Baseline must occur within 28 days of screening; otherwise, the participant must be re-screened to ensure continued eligibility. Once the baseline assessment is complete, randomisation occurs. The study interventionist assigned to the case communicates directly with the participant about their treatment assignment and schedules the first session. Participants are given 12 weeks after randomisation to complete all intervention sessions, which provides a realistic window to complete the intervention. The blinded research assistants conduct the follow-up assessments for each participant at 8 weeks (range 6–16 weeks), 20 weeks (range >16–28 weeks), and 32 weeks (>28–40 weeks) after randomisation. Table 1 displays the schedule of study procedures and Fig 1 displays the study flow.

### Sample size {14}

The sample size calculation to conduct a fully powered trial was informed by a meta-analysis of 22 randomised controlled trials evaluating digital psychological interventions in LMICs [18]. The meta-analysis found an average effect size of Hedge’s *g* = 0.60 in favour of the digital intervention, *g* = 0.53 for interventions addressing substance use, and *g* = 0.43 when an active control group was used. We therefore assumed a more conservative effect size of *g* = 0.40 in favour of mhGAP-Remote to calculate the sample size. This translates to a true mean difference of 2.4 points in the AUDIT score change between treatment arms from baseline to 8 weeks (range 6 to 16 weeks) follow-up. We assumed a pooled standard deviation (SD) of 6 [38, 39]. Thus, with an allocation ratio of 1:1, a sample size of 99 participants in each treatment arm is required for statistical power of 80% and a two-sided type I error of 0.05. Prior clinical trials by the study team within similar settings and populations (clinic-based recruitment, unhealthy alcohol use) indicate a roughly 10% attrition rate [36, 40]. However, we chose a more conservative estimate of 20% attrition resulting in a total sample size of *N*_planned_ = 248 (*n* = 124 per treatment arm). Prior to recruiting participants into the fully powered trial, we plan to pilot the study procedures with up to 10 participants.

### Recruitment {15}

Participants are recruited into the study from hospitals and nurse-led health centres in Butha-Buthe district. Patients undergoing general screening to enter the health facility (e.g., ongoing COVID-19 or tuberculosis screening) or those in the waiting areas are approached and verbal consent sought to ask screening questions. Prior to conducting formal screening, participants are told briefly about the study (e.g., treatment study for alcohol use in adults taking place over several months). Participants who are further interested are taken to a private space where the screening can be conducted. Recruitment fliers with study phone numbers, managed by research assistants and study coordinators, are posted in areas of high patient traffic where study staff are not based in order to actively advertise the study. Healthcare providers can also refer patients who they think would be eligible and interested.

## Assignment of interventions: allocation

### Sequence generation {16a}

After the baseline assessment, participants are randomly allocated to either the mhGAP-Remote or mhGAP-Standard intervention with an equal probability for assignment to each condition using an allocation table that was uploaded to REDCap. Block randomisation was conducted and stratified by sex (male vs. female) and age (< 50 years vs. ≥ 50 years). Sex was chosen as a stratification factor due to established differences in the biological processing of alcohol in male vs. female bodies [41], which often translates into higher alcohol consumption levels in men than in women [42]. Age was also chosen as a stratification factor due to older adults typically having less comfort with technology [43], which is pertinent to the interventions under investigation.

### Concealment mechanism {16b}

The allocation table was prepared by an independent statistician not involved in the study. The randomisation is conducted in REDCap using this pre-specified allocation table, which prevents study staff from anticipating the allocation sequence.

### Implementation of the randomisation {16c}

The study coordinator, who is not involved in intervention delivery, utilises REDCap to randomise participants to intervention arms. Specifically, after the baseline assessment is complete, the study coordinator enters the participant’s ID number in REDCap, alongside the participant’s sex and age, and clicks “randomise” to reveal the participant’s allocation. The allocation cannot be changed after this step is complete. The study interventionist assigned to the participant then directly informs the participant about which treatment arm they have been allocated to and schedules the first intervention session.

## Assignment of interventions: Blinding

### Who will be blinded {17a}

Providers of the intervention and participants are not blinded to study arm allocation. The trained research assistants who conduct study assessments with participants are blinded to the study arm. The study statistician is not a member of the study team, and, thus, is blinded to study arm allocation until the dataset is closed and formal unblinding of the data takes place in  the process of data analysis.

### Procedure for unblinding if needed {17b}

There is no procedure for unblinding, as we do not foresee any reason (e.g., safety concerns) that would require blinded team members to be unblinded during the course of the study.

## Data collection and management

### Plans for assessment and collection of outcomes {18a}

Once a participant consents to study participation, self-reported assessments are collected in REDCap via interview. Trained research assistants collect dry blood spots (DBS) to assess for PEth, medical records are accessed to collect data on liver enzymes and HIV viral load (subsample with HIV only) and data is entered into REDCap. If participant medical records indicate a test result of more than 30 days old, research assistants escort participants to the medical staff to have new tests taken. All primary, secondary, and exploratory outcomes are collected at all timepoints (see {12} for description of outcomes).

Data quality is promoted by training assessors on adherence to data collection protocols and rules of trial conduct, including proper data collection techniques. Additionally, all members of the study team have completed formal human subjects research training in social and behavioural sciences, which is consistent with Good Clinical Practice. The AUDIT assessment is recorded and independent raters code randomly selected recordings to determine level of performance and adherence to assessment protocols.

### Plans to promote participant retention and complete follow-up {18b}

Our team has several strategies in place to promote retention to study assessments. To increase the likelihood of remaining in contact with participants throughout the study period, we collect primary contact information, including name, phone number, and preferred methods and times of contact. Additionally, the name, phone number, and relationship for two secondary contacts are also collected, in the event we cannot reach participants directly. The research assistant sends an SMS to the participant's phone after they have consented to the study and while they are still in the presence of the participant to ensure all participants can receive SMSs on their phone prior to randomisation. In addition, participants’ subsequent study-related assessment is scheduled prior to leaving their current visit. Participants also have the option of completing the self-report components of the assessments telephonically, to accommodate travels and migrant workers. Lastly, participants are compensated for their transport time and effort to attend assessment visits, with a range of 50 ZAR (~ 2.60 USD) to 150 ZAR (~ 7.80 USD) per assessment, depending on distance travelled.

### Data management {19}

Data is collected and managed using REDCap, a customisable electronic data collection and management software, hosted at the University Hospital Basel, Switzerland. All data is entered directly into the electronic forms on REDCap, thus creating an electronic Case Report Form (eCRF). All endpoint data is collected on the REDCap app and synced to the server when an internet connection is available. Hard copy data collection is used in the event of electricity failure or tablet malfunction. REDCap offers a secure database platform with validation tools to limit data entry errors by restricting entered values to those in plausible ranges as well as pop-up notifications confirming that decline to answer responses are not data entry errors [44, 45]. After data are synced to the server, a team member assigned to the task reviews the entire REDCap data record for completeness. Regular reports are run on the data to ensure accurate and complete data. A data clarification request is directed to the field team to address any missing or implausible data.

### Confidentiality {27}

All research-related data (except consent forms and study locator information) are coded using unique participant ID numbers. All research data are stored in a secure location with limited access. eCRFs are collected on password-protected study tablets via REDCap and data synced to the server as soon as an internet connection is available. Only trained study team members have access to the REDCap project containing participant data with unique personal login details. REDCap is compliant with the European Union’s General Data Protection Regulation [46]. When not in use, the tablets are stored in a locked cabinet within a locked room at the coordinating centre (see “Composition of the coordinating centre and trial steering committee {5d}”). Identifiable participant information (consent forms, locator information forms) are stored separately from research records in a locked cabinet in a locked room. Only the Lesotho-based research team has access to this information. The document linking participant IDs to participant names is password-protected and stored on a password-protected hard drive and backed up on a weekly basis. Only fully de-identified data will be made public. Audio recordings of intervention sessions and AUDIT assessments are stored on the study team’s Microsoft Sharepoint or a similar platform (hosted by the University Hospital Basel), which is only accessible to authorised study personnel.

### Plans for collection, laboratory evaluation and storage of biological specimens for genetic or molecular analysis in this trial/future use {33}

Liver enzyme results and HIV viral load are collected as part of routine clinical care in the health facility. If recent liver enzyme tests or HIV viral load tests (for subsample with HIV only) are unavailable, participants are referred for a routine test within the clinic from which they are recruited. DBS specimens for PEth are collected by trained study team members. After collection, DBS specimens are left to dry for up to 24 h. Dried samples are then placed in individual plastic bags with two desiccant bags and a moisture indicator cardboard. Once they have been prepared, they are stored temporarily in refrigerators within the laboratory of Butha-Buthe district hospital. On a bi-weekly basis, samples are transported in cooler boxes at 4°C for biobanking at Seboche laboratory until they can be shipped to a laboratory where analysis can take place. These samples are destroyed after processing is complete.

## Statistical methods

### Statistical methods for primary and secondary outcomes {20a}

Primary and secondary analyses will follow CONSORT guidelines and intention-to-treat principles (i.e., all participants will be analysed according to their randomisation condition, regardless of whether they complete the assigned treatment). A flowchart will describe the inclusion and follow-up of participants by study arm. Baseline characteristics will be described by study arm with summary statistics, such as mean and standard deviation for normally distributed data, median and interquartile range for non-parametric continuous data, or number and percentage in the case of categorical indicators. No formal testing between arms will be performed. Outcomes will be described by arm using summary statistics.

For the primary analysis, we will employ a linear regression model on the outcome of difference in mean AUDIT scores from baseline to 8 weeks follow-up (range 6–16 weeks) using the arm allocation, stratification factors, and site (if sample size per site allows). Only participants who have an AUDIT score at the 8-week assessment will be included in this analysis. Superiority of mhGAP-Remote will be demonstrated if the lower bound of the 95% Wald confidence interval of the difference between the arms is superior to zero.

We also plan, a priori, to conduct three separate sensitivity analyses on the primary endpoint. These are as follows:

1. *Per protocol*. The per protocol analysis is defined as participants who attended at least one session of mhGAP-Standard or mhGAP-Remote. The per protocol analysis will allow for the evaluation of the intervention’s effects on participants who received at least a small dose of the treatment. Thus, only participants who completed at least one session of the intervention and who have an AUDIT score at the 8-week follow-up assessment will be included in this analysis. The analysis will then follow the primary analysis plan in using a linear regression model to predict difference in mean AUDIT scores from baseline to 8 weeks follow-up, using arm allocation, stratification factors, and site (if sample size allows) as independent variables.
2. *Recovery*. If participants no longer meet the threshold of “hazardous drinking” at the 8-week follow-up assessment for alcohol use based on AUDIT score (< 6 for women, < 8 for men), they will be considered “recovered.” Otherwise, participants will be categorised as “non-recovered” because they continue to demonstrate a high burden of alcohol use symptoms. Rates of recovery will then be compared between mhGAP-Remote and mhGAP-Standard. Only participants with an AUDIT score at the 8-week follow-up assessment will be included in this analysis. For this analysis, we will employ a logistic regression model with recovery (yes/no) as the outcome and arm allocation, stratification factors, and site (if sample size allows) as independent variables.
3. *Clinically significant and reliable change*. We will follow procedures outlined by Jacobson and Truax [47] for identifying clinically meaningful change in psychotherapy research. Means and standard deviations on the AUDIT from both clinical and non-clinical groups similar to the current population, based on a recent study in South Africa [48], will be used to establish the minimum amount of change needed that likely reflects true change in alcohol consumption (i.e., change that is not due to chance). Based on this calculation, a change of at least 8.4 points in the AUDIT score is the minimum required change. Participants would therefore need to decrease their total AUDIT score by 9 points to have clinically significant and reliable change. For this analysis, we will employ a logistic regression model with clinically significant and reliable change (yes/no) as the outcome and the same independent variables as in the primary analysis (i.e., arm allocation, stratification factors, and site [if sample size allows]).

For secondary analyses, we will evaluate changes from baseline in (1) AUDIT scores at the 20- and 32-week follow-ups and (2) changes in the biomarker PEth at 8-, 20-, and 32-week follow-ups. We will employ multilevel mixed effects regression models and control for clustering of variance within individuals over the repeated measures. For the AUDIT, we will use a normal distribution and identity link. For PEth, we will evaluate the data’s distribution and choose a suitable regression model and link function for analysis. Models will be adjusted for the arm allocation, stratification factors, and site (if sample size per site allows). We will report adjusted mean differences between treatment arms. Furthermore, we will compare the maintenance of “recovery” and clinically significant and reliable change at the 20- and 32-week follow-up assessments across the treatment arms.

### Interim analyses {21b}

We do not plan to conduct interim analyses as we do not have any safety or efficacy concerns about this trial.

### Methods for additional analyses {20b}

As part of the main analyses, we do not plan to carry out additional subgroup or adjusted analyses. However, we plan to conduct exploratory subgroup analyses by sex for the primary outcome. The analysis will be performed as the main primary outcome analysis and include an interaction term between sex and treatment allocation. If the interaction term is found to be significant, effect estimates will be summarised by sex. This will allow us to evaluate whether the intervention was differentially effective for men versus women. Given the established importance of sex and gender in health research [49], it is of a priori importance to us to evaluate possible differential effects of the intervention for men versus women. Additional analyses (e.g. mediator and moderator analyses, or analyses of baseline data) will also be conducted in an exploratory fashion rather than as part of main analyses described in this protocol. Such exploratory analyses will be disseminated in separate publications from main analyses.

### Methods in analysis to handle protocol non-adherence and any statistical methods to handle missing data {20c}

Previous clinical trials conducted by the study team have recorded less than 10% attrition [36, 40]. In the power analysis for the present study, we have used a conservative estimate of 20% attrition. Participants who drop out, who fail to attend a study assessment, or who are lost to follow-up will not be replaced by new participants, as we have accounted for this in the sample size estimate. Missing baseline and outcome data will be summarised by study arm and reported in the CONSORT. If attrition is higher than anticipated, multiple imputation will be used to impute the AUDIT score [50], and primary and secondary analyses will be conducted with the imputed datasets.

### Plans to give access to the full protocol, participant-level data, and statistical code {31c}

The authors plan to grant full public access to trial protocols as well as statistical code and underlying participant-level data for published analyses through the Open Science Foundation (OSF) repository for the study: https://osf.io/ftqyd/.

## Oversight and monitoring

### Composition of the coordinating centre and trial steering committee {5d}

The coordinating centre is located in SolidarMed’s field office in Butha-Buthe, Lesotho. This is a secure, locked building with secure, locked rooms inside of which the study team can safely store data. The trial steering committee consists of the study PIs (a clinical psychologist and a microbiologist), the Sponsor-Investigator (a medical doctor), a psychiatrist, and two PhD candidates in clinical research. Most steering committee members meet weekly to plan and manage the trial and discuss issues that arise. They will continue to meet weekly as the study is implemented and follow-up data collected. The trial steering committee oversees data management including collection and entry of data, plans day-to-day trial implementation and supervision for interventionists, oversees participant recruitment, and reviews and addresses safety events as they occur.

### Composition of the data monitoring committee, its role, and reporting structure {21a}

A Data Safety and Monitoring Board (DSMB) has been formed, comprising of three experts in clinical trials, biostatistics, behavioural interventions, and mental health in LMIC settings who are not involved in the study and who do not have scientific, professional, or financial conflicts of interest. The DSMB meets with the study investigators at the beginning of the trial and at regular intervals thereafter to review trial progress, as well as to review non-identifying reports of adverse events and steps taken by the study team to address them. The DSMB provides guidance about any further next steps that might help protect participant safety and well-being and ensure a high level of scientific compliance. The DSMB may also be contacted outside of planned meetings if the investigators believe their input is needed. DSMB members receive an honorarium for participating in each meeting.

### Adverse event reporting and harms {22}

Study staff interacting with participants are trained to understand what constitutes an adverse event (AE). Research assistants systematically inquire about any changes in participant physical or psychological well-being at each of the study’s follow-up assessment (i.e., at 8, 20, and 32 weeks). Recording of AEs outside of these assessments (e.g., assessment reminder phone call, intervention session) occurs on an ad hoc basis. Staff members record details of the AE; study coordinators and PIs are then made aware of the event. The study PIs evaluate the severity of the AE according to the Common Terminology Criteria for Adverse Events grading system [51] and make a causality assessment of the event in relation to the study intervention according to the standards set by the International Conference on Harmonisation, topic E2A [52]. Relatedness options of the study intervention to the AE are definitely unrelated, unlikely, possibly, probably, or definitely related.

In the event of a serious adverse event (SAE) that is “possibly,” “probably,” or “definitely” related to the study intervention, the event is reported to the ethics committee in Lesotho within 15 days of the study team learning of the event [53]. All other SAEs and AEs are reported to the ethics committee annually. Participants who terminate the study with reported ongoing SAEs that are “possibly,” “probably,” or “definitely” related to the study intervention are followed up until resolution or stabilisation of the SAE, for up to 6 months after study termination. If immediate safety and protective measures must be taken during the conduct of the study, the study PIs notify the ethics committee of these measures, and of the circumstances necessitating them, within 7 days. In the primary publication describing the trial results, a summary of all AEs will be provided according to severity and relatedness to the study intervention.

### Frequency and plans for auditing trial conduct {23}

We do not have plans for external trial auditing. Trial conduct and data are monitored via the trial steering committee and the DSMB, as described above. Yearly reports on study progress and trial conduct are sent to the ethics committee. If concerns arise regarding trial conduct, the study team may appoint an independent auditor to identify and resolve these concerns.

### Plans for communicating important protocol amendments to relevant parties {25}

Protocol amendments are decided on jointly by the study PIs and Sponsor-Investigator. Input from other study investigators and team members is sought to inform the decision for protocol amendments. Any protocol amendments must first be approved by the locally responsible ethics committee. Once approved, protocol amendments are communicated to the study team through email or routine meetings. As appropriate, changes are made to the study’s trial registration page at clinicaltrials.gov. Protocol changes that directly influence participants’ experience in the trial and which would also require a new consent form, are communicated to trial participants at their subsequent study visit, where the updated consent form would be signed.

### Dissemination plans {31a}

Findings of this study will be disseminated through peer-reviewed journal articles, conference presentations, and sharing of code, protocols, and manuscripts on open-access platforms such as OSF. The results of this trial will also be shared at relevant stakeholder meetings in Lesotho, such as via the Ministry of Health and community outreach, as well as internationally. Publication preference will be given to journals with an open access publication model. Study investigators will have the opportunity to contribute to the publication of results. To facilitate capacity building and foster academic careers in Lesotho, the study team encourages Basotho collaborators to contribute to publications resulting from this study.

## Discussion

The goal of this open-label randomised trial is to test the relative effectiveness of mhGAP-Remote versus mhGAP-Standard on alcohol use. The study uses a mix of self-report (primary) and biological (secondary) assessments to measure alcohol use, as well as exploratory assessments of the intervention’s effect on liver enzymes and HIV viral load (for subsample with HIV). The exploratory data on liver enzyme tests will provide biologically supportive evidence of the effect of alcohol consumption reduction on liver function. Data on HIV viral load will add to the limited data about whether behavioural interventions for alcohol use or other mental health problems translate into changes in HIV viral load [54–56]. Overall, information from this trial will provide data on the extent to which a primarily SMS-delivered intervention can effectively reduce alcohol use in a mainly rural LMIC setting, where very few human resources dedicated to mental health exist [57–59].

Although mhGAP-Remote is a novel intervention specifically developed for the current study, prior research has used SMSs to improve alcohol treatment effectiveness. One study in Uganda for people with HIV compared the effectiveness of booster sessions using either technology (SMSs or interactive voice recordings) to provider-delivered phone calls [19]. The study found that these interventions were more effective than standard of care (providing brief advice) on self-reported alcohol use, but not in the assessment using PEth. Several other studies have examined delivery of a primarily or completely SMS-based intervention after post-injury discharge from the hospital [22, 23] or after inpatient detoxification [21]. However, none of these studies tested the premise of using SMSs as an alternate to in-person provider-delivered care for alcohol use, which is the standard approach to delivering treatment. Nevertheless, these SMS-based interventions for alcohol use appear to be feasible and acceptable in sub-Saharan Africa, including in Uganda, Tanzania, South Africa, and Nigeria [19, 23, 24, 60]. These interventions also require fewer resources to deliver than standard interventions requiring in-person delivery, making them especially appropriate for delivery in LMIC settings [60]. The results of this study will therefore add to this fast-developing literature and inform future efforts to disseminate and scale-up effective interventions for alcohol use in low-resource settings.

This trial has strengths and limitations. With regard to strengths, the study uses a randomised design to assess an innovative intervention with high potential for scale-up and evaluates the outcomes using blinded assessors. Study limitations include limited external validity based on study selection criteria, which includes individuals who have regular access to a mobile phone. Moreover, the effectiveness of mhGAP-Remote depends on successful delivery of SMSs and active engagement by participants with the messages. Prior research with an SMS-derived intervention for alcohol use reports challenges with SMS delivery [23]. The system used to deliver the SMSs in the current study can register whether the message was sent correctly, but not whether the SMS was read. As a result, measuring objective adherence to the mhGAP-Remote intervention will be limited to participant self-report.

Overall, this study will inform the effectiveness of a brief, scalable, low-resource intervention to reduce unhealthy alcohol use. This study has the potential to inform practice and policy in Lesotho and other remote, resource-constrained environments by testing a combined in-person plus SMS-based intervention that can likely reach a greater number of people with fewer human resources.

## Trial status

Protocol version 1.0 (May 15, 2023).

We pilot tested our study procedures in August 2023 and began enrolment for this trial in September 2023. We anticipate completion of recruitment in April 2024.

### Supplementary Information

Supplementary Material 1: Supplementary Table 1. Intervention content in mhGAP-Standard. Note.

Supplementary Material 2: Supplementary Table 2. SMS content and schedule in mhGAP-Remote.

Supplementary Material 3. Example study consent form. 

Supplementary Material 4. SPIRIT guidelines checklist.

## Data Availability

A de-identified dataset used for analysis of primary, secondary, and exploratory outcomes will be made publicly available once the trial is concluded and the data has been analysed as planned by the authors. This dataset will be available for at least 3 years following the conclusion of the study on the trial’s OSF repository: https://osf.io/ftqyd/.

## Abbreviations

AE: Adverse event
ALT: Alanine aminotransferase
AST: Aspartate aminotransferase
AUDIT: Alcohol Use Disorders Identification Test
CONSORT: Consolidated Standards of Reporting Trials
DBS: Dried blood spot
DSMB: Data Safety and Monitoring Board
eCRF: Electronic Case Report Form
EKNZ: Ethics Commission of Northwest and Central Switzerland
HIV: Human immunodeficiency virus
LMIC: Low- and middle-income country
mhGAP: Mental Health Gap Action Programme
OSF: Open Science Foundation
PEth: Phosphatidylethanol
PI: Principal investigator
SAE: Serious adverse event
SD: Standard deviation
SMS: Short message service
WHO: World Health Organization
ZAR: South African Rand
