# Supplementary material for: SMSs as an alternative to provider-delivered care for unhealthy alcohol use: study protocol for Leseli, an open-label randomised controlled trial of mhGAP-Remote vs mhGAP-Standard in Lesotho
Source: Trials. 2024 Sep 2;25:575. doi: 10.1186/s13063-024-08411-3 (PMC11368011; doi:10.1186/s13063-024-08411-3)
Supplement: Supplementary file 1 — Supplementary Material 1: Supplementary Table 1. Intervention content in mhGAP-Standard. Note. [file 13063_2024_8411_MOESM1_ESM.docx]

Supplementary Table 1. Intervention content in mhGAP-Standard

|  | Session 1 | Session 2 | Session 3 | Session 4 | Booster 1 | Booster 2 |
| --- | --- | --- | --- | --- | --- | --- |
| Session agenda and home practice review |  | X | X | X | X | X |
| Introduction and overview to the program | X |  |  |  |  |  |
| Psychoeducation about alcohol use | X |  |  |  |  |  |
| Motivational interviewing to identify alcohol use goal | X |  |  |  |  |  |
| Motivation check-in |  | X | X |  | X | X |
| Identify alcohol use triggers |  | X | X |  |  |  |
| Strategies for reducing or stopping use or reducing alcohol use harms |  | X | X | X | X | X |
| Consolidate treatment gains and plan for future challenges |  |  |  | X |  |  |
| Decision about booster sessions |  |  |  | X | X |  |
| Session wrap-up | X | X | X | X | X | X |
| Home practice assignment | X | X | X | X* | X* |  |

Note. *Only if continuing with additional booster sessions.
