# Supplementary material for: SMSs as an alternative to provider-delivered care for unhealthy alcohol use: study protocol for Leseli, an open-label randomised controlled trial of mhGAP-Remote vs mhGAP-Standard in Lesotho
Source: Trials. 2024 Sep 2;25:575. doi: 10.1186/s13063-024-08411-3 (PMC11368011; doi:10.1186/s13063-024-08411-3)
Supplement: Supplementary file 2 — Supplementary Material 2: Supplementary Table 2. SMS content and schedule in mhGAP-Remote. [file 13063_2024_8411_MOESM2_ESM.docx]

Supplementary Table 2. SMS content and schedule in mhGAP-Remote

| Days after in-person session | Message content |
| --- | --- |
| 0 | Welcome to the Leseli program! You’ll receive 2 messages a week to support you on your alcohol use journey. |
| Week 1 (days 1 – 7) | |
| 1 | Does alcohol help you achieve some of your goals? What does alcohol prevent you from achieving that is important to you? Remember this for the whole week. |
| 4 | Identify your triggers. Name a person, place, or thing that makes you want to drink alcohol. Choose one and make a plan not to interact with it this week. |
| Week 2 (days 8 – 14) | |
| 8 | New ways of reducing alcohol consumption can be hard, but stick to it. |
| 11 | If you realize that you drink when upset, try to take a walk, watch TV, or talk with other people for at least 30 minutes. |
| Week 3 (days 15 – 21) | |
| 15 | Reducing or stopping alcohol use is possible. Everyone has their own alcohol use goal. What is YOUR goal? Remember this for the whole week. |
| 19 | Not keeping alcohol at home will make it easier to achieve your goal of reducing alcohol use. Try this plan of removing all available alcohol in the house. |
| Week 4 (days 22 – 28) | |
| 23 | Alcohol is a harmful substance. If you use too much of it, it can attack your liver, stomach, and brain. These effects can last a long-time. |
| 25 | What do you like to do while relaxing besides drinking alcohol? For example, sports or other things. Choose one you can do at least once a week. |
| Week 5 (days 29 – 35) | |
| 29 | It is not easy to reduce alcohol use. Remember how alcohol impacts your life. Does it affect your health, your work, or relationships with others? |
| 32 | What other things give you a sense of accomplishment besides alcohol? For example, cleaning or other things. Choose one activity you can do at least once a week. |
| Week 6 (days 36 – 42) | |
| 36 | Why is reducing or stopping alcohol consumption important to YOU? Write down your reason where you will see it every day. |
| 40 | Remember, there are ways to stick to your alcohol use goals. You learned skills and can use them anytime you feel you need them. |
| Week 7 (days 43 – 49) | |
| 44 | Do you find yourself drinking while with certain people or at certain places? Try to reduce contact with such people or places. |
| 47 | Many accidents are related to alcohol. The chance of being involved in an accident is reduced when you reduce your alcohol consumption. |
| Week 8 (days 50 – 56) | |
| 50 | Keep in mind what places, people or situations make you feel like drinking. Be aware of them even after finishing the program so you can stick to your goals. |
| 52 | Alcohol will be always available. How are you going to stick to your goals? Keep in mind the strategies you learned. You can do it! |
| 53 | You’ve now finished the Leseli program! You can refer back to these SMSs any time you need motivation or support in achieving your alcohol use goal. |
