# Supplementary material for: SMSs as an alternative to provider-delivered care for unhealthy alcohol use: study protocol for Leseli, an open-label randomised controlled trial of mhGAP-Remote vs mhGAP-Standard in Lesotho
Source: Trials. 2024 Sep 2;25:575. doi: 10.1186/s13063-024-08411-3 (PMC11368011; doi:10.1186/s13063-024-08411-3)
Supplement: Supplementary file 3 — Supplementary Material 3. Example study consent form. [file 13063_2024_8411_MOESM3_ESM.docx]

INFORMATION SHEET AND CONSENT FORM

**You are Being Asked to Be a Participant in the Study**

**“Substituting SMSs for Provider-delivered Care to Improve Alcohol Outcomes in People with and without HIV in Lesotho”**

**This research project is in collaboration between the following institutions and research investigators:**

-SolidarMed Lesotho: Malebanye Lerotholi (Local Principal Investigator)

-University Hospital Basel: Dr. Jennifer Belus (Principal Investigator) and Prof. Niklaus Labhardt (Chief Sponsor-Investigator)

-Ministry of Health Lesotho: Dr. Tun Shwe Kyaw and Pearl Letsoela (Co-Investigators)

**This Informed Consent Form has two parts:**

Part I: Information Sheet (to share information about the research project with you)

Part II: Certificate of Consent (for signatures if you agree to take part)

**PART I: Information Sheet**

**1. Introduction**

Hello, I am _____, working for this research project. I am speaking with you today to let you know about a study that we are doing that may help you use alcohol in a healthier way.

This information sheet is designed to tell you everything you need to think about before you decide if you want to be a part of the study. **It is entirely your choice. If you decide to take part, you can change your mind later on and withdraw from the research study.** The decision to join or not join the research study will not cause you to lose any medical benefits. If you decide not to take part in this study, any medical providers you have will continue to treat you. There may be some words in this information sheet that you do not understand. Please ask me to stop as we go through information and I will take time to explain it to you. If you have questions later, you can ask them to me or the contacts listed below

Before making your decision:

- Please carefully read this information sheet or have it read to you
- Please listen to the study staff explain the study to you
- Please ask questions about anything that is not clear

**If you agree to participate, you will be given a signed copy of this Informed Consent Form.**

**2. Purpose of the research project**

Alcohol use is common, but can cause serious health and quality of life problems for people who consume alcohol in an unhealthy way. For that reason, we want to provide better access to alcohol use treatment for people in the community. At the same time, there are very few trained counselors.

That’s why our team created a new treatment that uses mostly SMSs (the messages you receive on your phone) to help people adopt healthier drinking behaviors. We want to see if this treatment, which involves meeting with a trained counselor one time and follow-up SMSs, is a helpful program and if it is accepted in your community. To do this, we will compare this new SMS treatment to a version of the treatment that is provided completely in person (no SMSs) by a trained counselor.

**3. Who the study is for**

The study is designed for people living with or without HIV attending one of the selected health centers or district hospitals in Butha Buthe or Mokhotlong and plans to stay at the clinic for the duration of the study. To be eligible for the study, you must meet the criteria for “hazardous drinking”, have access to a cellphone at least half of the week and electricity to charge the phone, and be comfortable receiving study related SMSs on the phone. You must also be willing to participate in a study focused on problem drinking, be able to read and write in Sesotho or English (or have a treatment supporter able to read study-related materials), and willing to have intervention sessions audio-recorded.

**4. Procedures**

If you agree to participate, you will receive an individual baseline assessment. During this assessment, we will do the following:

- Ask you questions about yourself, including demographics (like age and employment), questions about your alcohol use and how you’ve been feeling, coping strategies, use of healthcare services, your social network and support, and quality of life. These will be recorded by a research assistant on an electronic device.
- We will also take a dried blood spot sample to detect the level of alcohol in your blood. This involves a small finger prick.
- Trained study staff will also access your medical records to get details of your HIV treatment history (if applicable), other information regarding your liver functioning, and healthcare use. If your HIV viral load or liver functioning tests are not available within the past 30 days, we will ask the nurse to take a blood sample.
- After the baseline assessment, you will be randomly selected by a computer program to receive treatment in one of two groups. Being randomly selected is like tossing a coin and the researchers do not have control over which group you end up in. You cannot switch groups once the randomization occurs.
- **mhGAP-Standard:** If you are selected into this group, you will be provided with an already existing intervention, developed by the World Health Organization, which has been shown to help people successfully reduce or stop their drinking. You will have 4 in-person sessions (45-60 mins each) with a trained counselor, with the possibility of up to 2 additional sessions. You will meet with the counselor individually.
- **mhGAP-Remote:** If you are selected into this group, you will be provided with a similar intervention as mhGAP-Standard, but the intervention is delivered mostly through standardized SMSs. You will attend one in-person session, which will be followed by approximately 8 weeks of SMSs that help you with the skills you learned in the first session. You can also speak to your counselor on the telephone to get additional support.
- Regardless of which group you end up in, the treatment will last for about 8 weeks. You will then complete additional assessments so we can see how you’re doing and determine whether the intervention was helpful. These follow-up assessments will be very similar to the baseline assessment you completed and will take place at approximately 8-, 20- and 32-weeks after your enrolment in this study.

**5. Audiotaping**

The intervention sessions will be audio-recorded in order for us to make it easier to remember what you said and to make sure that the correct procedures are being followed by the interventionist. No one other than a research staff member will be allowed to listen to the tapes. The audio recordings will be stored safely and securely on a password-protected shared network, with access only by research staff working on this study.

**6. Duration**

The active intervention phase is expected to take place over approximately 8 weeks, but no more than 12 weeks. After this time, the study-related interventions will end. You will then complete three more follow-up assessments, which will take place at approximately 8-, 20-, and 32-weeks after your enrolment in this study. For these follow-up assessments, we will ask you to visit the health facility to complete the assessments in person.

**7. Risks/discomforts**

There are a few risks to taking part in this study. First, you might feel emotionally upset during the interview or the intervention. It is okay if you do not want to talk about certain topics or answer certain questions. Study assessors and interventionists will be trained in how to deal with negative emotions. Second, there is also the chance that a breach (or lapse) in confidentiality may occur and participants’ health-related data is inadvertently revealed to individuals outside of the study. However, we plan to reduce this possibility in several ways, such as making sure that all staff receive training in research ethics and data confidentiality, password protecting any identifiable information (like your name), and using a unique identification (ID) number assigned to your data, which will only be known to research staff on the study.

A third risk in the study is if you have your blood drawn via a dried blood spot, there may be bleeding, swelling, or bruising at the site of the blood draw. Should any of these issues arise, trained personnel taking blood will advise on the appropriate actions to take. We will help you seek additional medical care, if needed.

Finally, there is a low risk of worsening of alcohol symptoms and/or physical harm due to dangerous withdrawal symptoms. We will give you a list of dangerous withdrawal symptoms to be aware of. If any of these occur, we help you seek immediate medical management. Study assessors will also monitor your alcohol intake during the study.

**8. Benefits and Reimbursements**

We cannot guarantee that you will receive any direct benefit from participating in this study. Your participation is likely to help us find answers to the important research question of how to help increase access to alcohol use treatment in Lesotho and other similar settings. It is possible that you may notice improvement in your alcohol use and/or improvement in engagement in HIV care (if applicable), but we cannot guarantee this.

In return for your time participating in the research, you will be reimbursed with ZAR 50 to 150 cash for each assessment you complete (baseline, 8-weeks, 20-weeks, and 32-weeks follow-up), to compensate you for your time and travel costs. You will not receive any compensation for attending the intervention sessions.

**9. Voluntary Participation and Right to Withdraw**

Your participation in this research project is entirely voluntary and you may withdraw from the study at any time without losing any of your rights as a patient. If you decide to discontinue participation, it will not affect your medical assistance and other treatment options available to you in any way.

You may also withdraw your consent in the future. If you decide to do so, you can also tell us if you would like to withdraw any data that has already been collected. In the event that your data has already been used in publications, it cannot be removed. However, all future use of your data will be removed.

In the event that your alcohol use significantly worsens during the course of the study such that you require a higher level of care, or the study investigators believe your participation in the study causes a danger to you or others, you can be removed from the study.

**10. Confidentiality**

The information that we collect in this research project will be kept confidential. This means that information you share with us will not be shared with others outside of the study research team. The only time we would share your information with others outside of the study is in one of the following situations: (1) you tell us that you might harm yourself, (2) you tell us that you might harm someone else, or (3) you tell us that a minor is being abused. Otherwise, any information we collect about you will be confidential and safely stored.

Only trained staff will have access to your information. Your name will not be mentioned on the data collected during the study. You will be given a unique identification (ID) number, which will be used to identify data collected. Only the researchers will know what your number is and we will keep that information protected. If the results of this study get published in a scientific journal, your name will not appear on the publication. However, if improper disclosure does occur, you will be notified immediately. In order to ensure good data quality, a competent authority or an ethics committee may require direct access to the data collected in this study.

**11. Who to Contact**

If you have any questions, you may ask them now or later, even after the research project has started. If you wish to ask questions later, you may contact the following person of the research team at any time:

Malebanye Lerotholi, Study Principal Investigator, SolidarMed Lesotho, +266 630 15586

Mokhali Mokhu, Study Coordinator, SolidarMed Lesotho, +266 585 40346

**PART II: Certificate of Consent**

I have read this informed consent form (or have had it read to me) and I have had the opportunity to ask questions about the research study. These questions have been adequately answered. I therefore understand the purpose of this research study focused on reducing alcohol use and I understand the following circumstances:

- I understand that I am participating freely and without being forced in any way to do so.
- I understand that I can stop participating at any point should I not want to continue and that this decision will not, in any way, affect me negatively.
- I understand that this is a research project, which may not benefit me personally in the immediate or short-term.
- I understand that the intervention sessions for mhGAP will be audio-recorded, stored electronically, and will be used for research purposes now or at a later stage.
- I understand that I am giving the researchers access to my medical records to allow them to extract my medical data related to my health and healthcare use.
- I understand I may be asked to leave the study before it has finished if the researcher believes it is in my best interest or if I do not follow the study plan as agreed to.

**I hereby agree to participate in the study on** **Substituting SMSs for Provider-delivered Care to Improve Alcohol Outcomes in People with and without HIV in Lesotho**.

| ___________________  Participant name | ___________________  Signature of participant | _______________  Date |
| --- | --- | --- |
| __________________  Staff member name | ____________________  Signature of staff | ________________  Date |

If participant is illiterate, please have them mark their thumbprint in the box below and have a witness write their name, signature, and date.

Participant thumbprint

| ___________________  Witness name | ___________________  Signature of witness | _______________  Date |
| --- | --- | --- |

**LEQEPHE LA LITABA LE FOROMO E FANANG KA TUMELLO**

**U kopuoa hore u nke karolo boithutong ba ts’ebeliso ea melaetsa ea thekeng e mabapi le phano ea lits’ebeletso tsa ntlafatso ea ts’ebeliso ea tai ho batho ba phelang le kokoana-hloko ea HIV le ba sa pheleng le eona ‘Substituting SMSs for Provider-delivered Care to Improve Alcohol Outcomes in People with and without HIV in Lesotho'**

**Morero ona oa lipatlisiso o tsamaisoa ka ts’ebelisano ‘moho le litsi tse latelang le bafuputsi ba lipatlisiso:**

-SolidarMed Lesotho: Malebanye Lerotholi (Local Principal Investigator)

-University Hospital Basel: Dr. Jennifer Belus (Principal Investigator) and Prof. Niklaus Labhardt (Chief Sponsor-Investigator)

-Ministry of Health Lesotho: Dr. Tun Shwe Kyaw and Pearl Letsoela (Co-Investigators)

**Foromo ena e kopang tumello e na le karolo tse peli:**

- Karolo I: Leqephe la Litaba (ho u arolela litaba ka morero oa lipatlisiso)
- Karolo II: Lengolo la Tumello (bakeng sa motekeno haeba u lumela ho nka karolo)

**KAROLO I: Leqephe la litaba**

**1.Selelekela**

Lumela Ntate/Mme ke ____________________________, ea sebeletsang morero ona oa lipatlisiso. Ke tlo bua le uena ka boithuto bo ka u thusang ho sebelisa tai ka mokhoa o nepahetseng.

Leqhephe la litaba le etselitsoe ho u fa ntho engoe le engoe eo uka lakatsang ho e tseba pele u nanaha hore u ka nka karolo boithutong. **Ke khetho ea hau ka botlalo ha u etsa qeto ea ho nka karolo, u ka fetola monahano oa hau ha morao kapa oa tsoa boithutong ha morao.** Haeba u khetha ho nka karolo kapa u khetha ho se nke karolo boithutong, u ka se lahleheloe ke litokelo tsa hau u le mokuli. Haeba u etsa qeto ea ho se nke karolo boithutong bona, bafani ba tsa bophelo ba tla tsoela-pele ho u alafa. Ho kaba le mantsoe a mang leqhepheng la litaba ao usa a utloisiseng ke tla kopa utlo re ke eme ha re ntse re tsoela-pele ka litaba ‘me ke tla nka nako ho u hlalosetsa. Haeba u kaba le lipotso ha morao, u tla mpotsa tsona kapa oa letsetsa linomoro tse thathamisitsoeng ka tlase.

Pele u ka nka qeto:

- Ka kopo bala leqhephe la litaba kapa u le balloe ka hloko
- Ka kopo mamela basebetsi ba boithuto bao thlalosetse ka boithuto
- Ka kopo utlo botse lipotso ka ntho e ngoe le e ngoe esa thlakang

**Haeba u lumela ho nka karolo, utla fuoa foromo ea tummello e tekennoeng**

**2. Sepheo sa morero oa lipatlisiso**

Ts’ebeliso ea joala e tloaelehile empa eka baka mathata a tebileng abophelo bo botle ho batho ba sebelisang tai ka tsela esa nehapalang. Ka lebaka lena re batla ho fihlela tse’beliso e betere ea tai le pheko ho sechaba sa heno. Ka tsela e t’soanang hona le bahlabolli ba fokolang ba rupetsoeng.

Ke ka hoo sehlopha sa rona se entseng pheko e ncha e sebelisang melaetsa (melaetsa eo u e fumanang mohaleng oa hao). Re batla ho bona haeba pheko ena e kenyellatsang ho kopana le mohlabolli ea koetlisitsoeng ha ngoe le ha uso tla tlhabollong ea morao ea melaetsa etla thusana ‘me etla thabeloa ke sechaba sa heno. Ho etsa sena re tlo bapisa melaetsa e mecha e sebelisoang ea pheko le pheko e etsoang ha motho a itlisitse ka seqo a kopana le mohlabolli ea koetlisitsoeng ho sena melaetsa.

**3.Khetho ea ho nka karolo boithutong**

Boithuto bona bo entsoe bakeng sa batho ba phelang le kokoana-hloko ea HIV, le ba senang HIV ba fumanang kalafo litsing tsa bophelo tse hloauoeng, kapa lipetlele tsa Botha-Bothe le Mokhotlong, batlang ho etsetsa lit’shebeletso tsa bona setsing sa bophelo seo ho fihlela boithuto bo felile. Hore u nke karolo boithutlong, u tlameha u bo sebelisa tai ka tsela e kotsi, u bona le motlakase ho chaja(charge) mohala oa thekeng hore utlo nno fumane melaetsa mohaleng oa hao. U bona le khahleho ea ho nka karolo boithutong bona, u khona ho bala le ho ngola Sesotho le Sekhooa (kapa ho ba le motšehetsi oa phekolo ea khonang ho bala lisebelisoa tsena).

**4. Tsamaiso**

Ha u lumela ho nka karolo: u tla fuoa tlhahlobo ea motheo ea motho ka mong. Nakong ea tlhahlobo ena, re tla etsa tse latelang:

- Re tlo u botsa lipotso ka uena, ho kenyelletsa le lilemo tsa hau le moo u sebetsang teng, lipotso ka t’sebeliso ea tai le maikutlo a hao le maano ao u a sebelisang ha u kopana le boemo bo itseng.
- Re tla nka mali re shebe boemo ba tai maling a hao. Ho kenyelletsa le ho hlajoa ha monoana.
- Basebetsi ba koetlisitsoeng batla hlahloba bukana ea hao ho fumana litaba ka botlalo mabapi le kalafo ea HIV, le hore na sebete se sebetsa joang. Haeba liphetho tsa sekhahla sa kokoana hloko le tsa t’sebetso ea sebete li le sieo ka matsatsi a mashome a mararo a fetileng, re tla kopa mooki ho kha mali ao a joalo.
- Ka mora tlhahlhobo, mochini oa computer o tla khetha ka lotho le ho arola batho ka maquloana a mabeli. Mochini etla ikhethela lequloana leo otla thusoa o le ho lona, ha una tokelo ea ho fetolela lequloaneng le leng.
- mhGAP- standard: Haeba u khethuoe sehlopheng sena, utla fuoa mokhoa tsamaiso oo le tlang ho fumana kalafo e entsoeng ke ba lefapha la World Health Organisation, o thusang batho ho fokotsa kapa ho emisa tai. Ho tla ba le mekhahlelo e mene ea tlhabollo kapa e tseletseng ea motho ka mong moo utla hlabolloa ke mohlabolli ea koetlisitsoeng nakong e ka etsang metsotso e mashome a mane a metso e mehlano ho isa ho hora.
- mhGAP-Remote: haeba u khethuoe sehlopheng sena, u tla nne u tsamaee methati e t’soanang le ea mhGAP-Standard, empa mona t’sebetso boholo etla etsoa ka mokhoa ho romela melaetsa mohaleng oa hau. Ho tla ba le tlhabollo ea motho ka mong e le ngoe feela etla lateloa ke beke tse robeli tsa ho fumana melaetsa etla u thusa ka mahlale ao u ithutileng oona ho tsoa tlhabollong ea pele. U ka bua le mohlabolli oa hau ka mohala ha ho hlokeha a ofe t’sehetso.
- Ho sa tsotellehe hore na u kene sehlopheng se feng, kalafo etla nka beke tse robeli, motho o tla nkisoa tlhatlhojoana ele ho sheba na seo a ithutileng sona o fela a atlehile ho una tse molemo kapa chee, tlhahlobo tsena li tla nka beke tse robeli ho isa ho tse mashome a mararo a metso e ‘meli kamora ho ba karolo ea boithuto.

**5. Khatiso ea mantsoe**

Lipuisano tsa motho ka mong li tla hatisoa. Molemo oa ho hatisa mantsoe ke ho re nolofaletsa ho hopola seo u se buileng le ho thuisa litaba tseo uena le ba bang le re fang tsona. Ha hona motho e mong ea tla lumelloa ho mamela likhatiso tsena ntle le mosebeletsi oa lipatlisiso. Leha ho le joalo re tla u kopa tumello pele re ka etsa joalo. Bobeli likhatiso le tse ngotsoeng pampiring li tla bolokoa ka t’sireletso le polokeho moo li tla fumanoa feela ke basebeletsi ba lipatlisiso ba sebetsang boithutong bona.

6. **Nako**

Ho kena lipakeng hoa mohato ona ho lebelletsoe ho nka nako e ka bang beke tse robeli, empa esa fete beke tse leshome le metso e ‘meli. Kamorao ho nako eo, mehato e amanang le boithuto etla emisa. Ebe u tla qetela tlhabollo ea morao-rao etla nka beke tse robeli ho isa ho tse mashome a mabeli le mashome a mararo a metso e ‘meli kamora ngoliso boithutong. Bakeng sa litherisano tse latelang, re tla u kopa hore u phethe tlhahlobo ea tatellano setsing sa bophelo ka seqo.

7 **Likotsi**

Kotsi tsa ho nka karolo boithutong bona lia fokola. Ea pele u ka ba le khatello ea maikutlo kapa ho t’soenyeha. Ha u hloke ho araba lipotso tse etsang u ikutloe u sa phutholoha. Ho lokile haeba u sa batle ho bua ka lihlooho tse itseng kapa tsa botho ba hau. U ka phomola nako eohle. Re tla u fetisetsa bakeng se seng moo u tla fumana thuso haeba u ikutloa u sa phutholoha ka sehlooho se ka qoqoang nakong ea lipuisano. Haeba u ikutloa u t’soenyehile nakong ea lipuisano kapa kamorao, re tla u fetisetsa ho mohlabolli.

Ea bobeli, makunutu a motho ea nkang karolo boithutong a ka qetella a tsejoa ke batho ba sa nkeng karolo boithutong. Le ha ho le joalo, re leka ho qoba seo ka tsela tsohle, ka hore basebetsi ba fumane koetliso ka melao ea boitsoaro ea lipatlisiso, ho boloka lekunutu le ho sireleletsa litaba ka ho kena ka mabitso a itseng a tsejoang ke batho ba koetlisitsoeng feela

Kotsi ea boraro boithutong ekaba hore nakong eo u khileng mali a omisoang pele e ea labong, u ka tsoa mali, ho ruruha kapa ho phumoha lehlakoreng le khuoeng mali. ‘Me ha e ngoe ea mat’soao ana a hlaha, ea koetlisitsoeng u tla u joetsa ka mehato eo u ka e nkang. Re tla u thusa ho fumana tlhokomelo e kenyelletsang ho bona ngaka ha ho hlokahala.

Ea ho qetela, kotsi ekaba eno ea ho mpefala hoa litla-morao tsa tai kapa ho itematsa ka lebaka la mat’soao a ho ikhula taing.

**8. Melemo le lipuseletso**

Ha rena netefatso ea hore hona le molemo o tlang ho uena ka kotloloho ha u nka karolo boithutong bona. Ha u nka karolo, u ka re thusa ho fumana likarabo tsa bohlokoa tsa lipatlisiso hore na kalafo ea t’sebeliso ea tai eka anetsoa ka hara naha ea Lesotho le libakeng tse ling tse t’soanang. Ho ka etsahala hore u bone ho ntlafala t’sebelisong ea hau ea tai le ho ntlafala tlhokomelong ea HIV (haeba e amana), empa ha rena netefatso ea taba ena.

Molemong oa nako ea hau lipatlisisong tsena, utla putsoa ka M50.00 ho isa ho M150.00 ha u qetile mokhahlelo ka mong oa lipotso (qalong, khoeli tse peli,khoeli tse hlano le likhoeli tse robeli tsa t’salo-morao) ho leboha nako ea hao le ea litjeho tsa maeto. Ha una fumana meropotso emeng ka lebaka la ho kena boithutong bona

**9. Ho nka karolo ka boithaopo le tokelo ea hao ea ho ikhula boithutong**

Ho nka karolo boithutong bona, ke boithaopo mme ka hona uka ikhula boithutong ka nako eo u batlang ho etsa joalo ka ntle ho lahleheloa ke litokelo tsa hau tsa ho fumana lit’sebeletso. Ha u etsa qeto ea ho se tsoele pele ka boithuto, lit’sebeletso tsa hau tsohle tsa bophelo li ke ke tsa ameha. U ka hula tumello eo u ileng oa fana ka eona. Ha u etsa qeto e joalo, u ka re tsebisa ha u lakatsa hore lipalo-palo tseo re seng re li bokelletse re li hule kapa che. Boemong boo lipalo-palo tsa hau se li kenelletse liphatlisisong li ke ke tsa huloa. Empa, liphatlalatsong tse tla etsahala ka mora hore u ikhule re tla hula lipalo-palo tsa hau.

Ha hoka etsahala hore t’sebeliso ea hau ea tai e mpefale nakong ea boithuto bona, mme eba u ka hloka tlhokomelo e ntlafetseng, kapa ebe mofuputsi oa boithuto o lumela hore ho nka karolo boithutong ho etsa kotsi ho uena kapa bathong ba bang, u tla huloa ka hara boithuto.

**10. Lekunutu**

Lintla tse tla bokelloa boithutong bona li tla bolokoa ele lekunutu. Sena se bolela hore lintlha tsohle tseo utla li arolelana le batho ba tsamaisang boithuto, ha lina aroleloa mang kapa mang eo eseng karolo ea boithuto bona. Nako eo re ka arolelang lintlha tsa hau le batho ba bang beo eseng karolo ea boithuto ke ha (1) u re bollella hore uka nna oa itematsa. (2) ha u re bollella hore o ka nna oa lematsa motho emong. (3) ha u re bolella hore motho ea tlase lilemong o oa hlekefetsoa. Ha hose joalo lintlha tsohle li tla bolokoa ele lekunutu li bolokoe sebakeng se bolokehileng.

Ke feela batho ba koetlisitsoeng ba tla fihlela lintlha tseo utla li arola le basebeletsi ba boithuto. Lebitso la hau ha le na sebelisoa, empa lintlha tsa hau li tla phatlalatsoa ka tsela e patileng mabitso a hau. Tsela ena e patiloeng ke e tla utluisisoa ke basebeletsi ba boithuto feela. Ha lipalo-palo tsa boithuto li phatlalatsoa lingoloeng tsa mahlale, lebitso la hau ha lena phatlalatsoa. Empa, ha ho kaba le taba e ka phatlalatsoang u tla tsebisoa hang-hang. Molemong oa ho netefatsa lipalo-palo motho ea nang le boiphihlelo ea fuoeng tumello kapa ekaba komiti ea boits’oaro li ka nna tsa hloka ho fumana litaba tse amanang le lintlha tsa hau tsa boithuto.

**11. Bao uka ikopanyang le bona**

Haeba una le lipotso uka botsa hona joale kapa hamorao, le kamora hore lipatlisiso li qale. Haeba u batla ho botsa hamorao, uka ikopanya le batho ba latelang ba moifo oa lipatlisiso nako eohle

Malebanye Lerotholi, Study Principal Investigator,SolidarMed Lesotho,

+266 630 15586

Mokhali Mokhu, Study Coordinator, SolidarMed Lesotho, +266 58540346

**Karolo ea II: Tiiso ea tumellano**

**ART number (if applicable):**

Ke balile, kapa ka ballooa taba tsa foromo ena. Ke bile le monyetla oa ho botsa litaba ka boithuto bona. Potso li arabetsoe hoo ke khotsofetseng. Ke utloisisa sepheo sa boithuto ba ho sebetsana le ho fokotsa t’sebeliso ea tai mme ke lumela tse latelang:

- Ke nka karolo ntle le khatello ea letho mme ke etsa hona ka bolokolohi.
- Kea utloisisa hore nka emisa ho nka karolo boithutong bona ka nako eohle ka khetho eaka. Mme qeto eno eaka ha ena ba le litla-morao life kapa life t’sebeletsong tse ke lifumanang.
- Kea utloisisa hore hona ke boithuto feela mme bo ka sebe le melemo e tlang ho nna ke le motho ha joale kapa ha morao.
- Kea utloisisa hore lipuisano tsa mhGAP li tla hatisoa, li bolokoe marang-rang mme li sebelisoe feela bakeng la boithuto ha joale le nakong e tlang.
- Kea utloisisa hore ke lokolla bafuputsi ba boithuto bona ho sheba/sebetsana le taba tsaka tsa bophelo le t’ sebeletso eaka bophelong ka kakaretso.
- Kea utloisisa hore nka koptjoa ho emisa boithuto bona pele bo fela ha feela baithuti ba bona bose molemong oa ka kapa ke sa latele litumellano tsa boithuto bona.

Mona ke lumela ho nka karolo boithutong ba “***Substituting SMSs for Provider-delivered Care to Improve Alcohol Outcomes in People with and without HIV in Lesotho”***.

| ___________________  Lebitso la motho ea  nkang karolo | ___________________  Motekeno oa motho ea  nkang karolo | _______________  Letsatsi |
| --- | --- | --- |
| __________________  Mosebeletsi oa boithuto | ____________________  Motekeno oa mosebeletsi | ________________  Letsatsi |

Ha motho a nkang karolo a sa tsebe ho bala le ho ngola, O taka ka monoana o motona ka (ink), a nto hatisa ka lebokoseng. Paki e tsebang ho bala le ho ngola (e khethuoeng ke motho ea nkang karolo) o tlameha ho tekena le eena.

Khatiso ea monoana

| ___________________  Lebitso la paki | ___________________  Motekeno oa paki | _______________  Letsatsi |
| --- | --- | --- |
